# Refinement of a training concept for tutors in problem-based learning

**DOI:** 10.3205/zma001115

**Published:** 2017-10-16

**Authors:** Konstanze Vogt, Jörg Pelz, Andrea Stroux

**Affiliations:** 1 Charité Medical University Berlin, Dieter Scheffner Centre for Higher Medical Education, Vice Deanship of Studies and Education, Berlin, Germany; 2 Charité Medical University Berlin, Dieter Scheffner Centre for Higher Medical Education, Berlin, Germany; 3 Charité Medical University Berlin, Institute of Biometry and Clinical Epidemiology, Berlin, Germany

**Keywords:** problem-based learning, tutor, training concept, interactive, interdisciplinary

## Abstract

The use of problem-based learning (PBL) in the Charité Berlin Human Medicine model curriculum requires the annual training of 80 to 100 new PBL tutors using PBL tutor training (PTT). Therefore, the following three measures were taken:

1. The existing traditional PTT (Trad-PTT) was further developed into an interactive PTT (Inter-PTT), which is using more interactive teaching tools. Both PTT concepts ran for 12 months, respectively. The review of the Inter-PTT was significantly better, as the PBL tutors understood their tasks within the PBL process better and felt more motivated. A follow-up survey after the initial experience with PBL confirmed almost all the positive aspects of the Inter-PTT.
2. In addition, the Inter-PTT was also offered to non-clinicians and other scientific staff to make the training interdisciplinary. PTT made it possible to communicate beyond specialist boundaries; however, the interdisciplinary idea was no longer detectable in the follow-up survey.
3. In order to increase the number of available PBL tutors, a self-commitment was introduced for the departments.

This increased the number of “involuntary” participants for PTT, but reduced the amount of necessary training courses. The fulfilment of self-commitment succeeded in almost all departments. A PTT tailored to the needs of the tutors is a basic prerequisite in order to excite teachers about PBL and to familiarise them with their role as learning facilitators. The increase of interactive teaching forms led to a joint interdisciplinary learning process within PTT. The excellent review of Inter-PTT makes it a solid basis for further training concepts.

## Zusammenfassung

Der Einsatz von Problem- orientiertem Lernen (PBL) im Modellstudiengang Humanmedizin der Charité Berlin erfordert die jährliche Schulung von 80- 100 neuen PBL Tutoren mittels PBL- Tutortraining (PTT). Deshalb wurden drei Maßnahmen ergriffen:

1. Das bisher durchgeführte traditionelle PTT (Trad- PTT) wurde zum interaktiven PTT (Inter- PTT) weiter entwickelt, das verstärkt mit interaktiven Lehrformen arbeitet. Beide PTT- Konzepte liefen jeweils 12 Monate lang. Die Bewertung des Inter- PTT war signifikant besser, die PBL Tutoren verstanden ihre Aufgaben im PBL- Prozess besser und fühlten sich stärker motiviert. Eine Nachbefragung nach den ersten Erfahrungen mit PBL bestätigte fast alle positiven Aspekte des Inter- PTT.
2. Zusätzlich wurde das Inter- PTT auch für Nichtkliniker und andere wissenschaftliche Angestellte angeboten, um das Training interdisziplinär zu gestalten. Die Kommunikation über Fachgrenzen hinaus gelang beim PTT; allerdings war der interdisziplinäre Gedanke bei der Nachbefragung nicht mehr nachweisbar.
3. Um die Zahl der verfügbaren PBL Tutoren zu erhöhen, wurde eine Selbstverpflichtung für die Abteilungen eingeführt.

Dies steigerte die Teilnahme “unfreiwilliger” Teilnehmer am PTT, reduzierte aber die Zahl der erforderlichen Schulungen. Die Erfüllung der Selbstverpflichtung gelang fast allen Abteilungen. Ein auf die Bedürfnisse der Tutoren zugeschnittenes PTT stellt eine Grundvoraussetzung dar, um Lehrende für PBL zu begeistern und mit ihrer Rolle als Lernförderer vertraut zu machen. Die Vermehrung interaktiver Lehrformen führte beim PTT zu einem gemeinsamen interdisziplinären Lernprozess. Die sehr gute Bewertung von Inter- PTT macht es zur Basis für weitere Schulungskonzepte.

## 1. Introduction

Problem-based learning (PBL) was introduced at the McMaster University in 1968 and is one of the most well-established teaching methods in medical curricula [1]. In contrast to teacher-centred lectures with passive learning and focus on exams, PBL represents a student-centred process of active learning in the sense of cognitive psychology [2]. PBL tutors play a key role by stimulating student learning, promoting teamwork within the PBL group, providing support for self-responsible learning, and providing feedback [3].

Until 2009, the Charité University Hospital in Berlin offered a traditional medical curriculum as a regular study course for 600 students annually. At the same time, a reform curriculum for 63 students was established in 1999 as an experiment (see Table 1 (Tab. 1)). The reform study course used PBL in organ-related modules [4]. The knowledge gained by the students in both courses was regularly checked using a progress test and resulted in a comparable cognitive level [5], as confirmed by Schmidt [6] and various US universities [7], [8]. The reform curriculum showed the already known positive effects: better professional competences, increased psychosocial skills and autonomous management of self-controlled learning [9]. 

The model curriculum for medicine, which has been binding for all students since 2010, combined traditional and reform curriculum with PBL as a standard teaching format. The planning of 40 PBL groups per semester required 80-100 new PBL tutors annually.

During the course of the reform curriculum (1999-2009), the PBL tutors had volunteered for training and teaching. All of them had experience as teachers and were convinced of this didactic method. Four times a year, an established PBL tutor training (PTT) was held in two variants: Clinicians received 2x8, non-clinicians 3x8 training modules. The three-day PTT had the same content but offered more time for the clinical context. After the PTT, the tutors sat in on someone else’s PBL lecture before they took over their own group. Only as many participants were admitted to the PTT as there are students in a PBL group in order to effectively communicate group dynamics in PBL. 

The situation is different in the model curriculum: In 2011, 37 PTTs were needed for 239 new PBL tutors, and in 2012 33 PTTs were required for 253 PBL tutors. In addition, the PTT instructors teach the model curriculum without sitting in on someone else’s lecture first. Soon there were complaints about the traditional PTT (Trad-PTT), because the tutors did not feel adequately prepared for problems in the PBL process. They considered the pedagogic and didactic content too theoretical and felt that it was missing references to deal with difficult students. 

Therefore, the PTT concept for the interactive PTT (Inter-PTT) was revised. The theoretical input was reduced by sending five articles about PBL before the PTT in order to arouse the interest of PBL-inexperienced teachers and to shorten the theoretical part in the actual PTT. This mail, which was sent two weeks before the training, served at the same time as a reminder about the PTT. 

The number of interactive teaching units has increased from 25% to 62.5% of the total time. While Trad-PTT problems were mainly mediated frontally in the group and with individual students, this proportion was reduced through group discussions (e.g. the perception of the tutor by the students or the balancing of permissive and directives in the classroom) as well as role playing (e.g. dealing with aggressive students and de-escalation strategies). The high proportion of interaction was supposed to teach the tutors that PBL does not require the front-line facilitator, but the facilitator himself. This role change was practised repeatedly because it was difficult for most of the tutors.

PBL case training was intensified by not only moderating individual PBL steps (as with Trad-PTT), but telling each tutor to introduce an entire PBL case (see Table 2 (Tab. 2)). While in Trad-PTT a non-medical case was discussed on the first day and a medical case on the second day, in Inter-PTT the tutors all received a PBL case on the second day, which was tailored to their own subject. These cases were derived from the foundations of proven PBL cases, i.e. there were also enough theory-oriented cases (for example, the calculation of the probability of cancer caused by UV radiation, the medical consequences of work-related diseases). The presentation of the PBL case in front of the other tutors, who represented the PBL group, revealed very quickly in the feedback whether the role as a facilitator had been successful. Many participants already recognized in their own reflections that they were too much involved in the discussion.

These three key elements produced the new concept, which started in 2013. It was more tailored to the needs of the tutors and was supposed to train them to develop a higher problem awareness and to control the learning process of the students [10]. The tutors should become a positive role model for the students. An important concern of Inter-PTT was to teach the tutors the learning process at PBL and to make them aware of their changing roles in the various PBL steps [11].

Clinical and non-clinical tutors were taught Inter-PTT jointly, because they were supposed to learn to promote the learning process as a facilitator instead of primarily imparting knowledge [12], [13]. Whether expertise is important in PBL is controversial, since PBL tutors, who are experts in the field, find it difficult to withdraw from the role as the facilitator [14]. However, expertise in the field offers advantages if the students have further questions [15]. Since the PBL evaluation did not show any differences between the assessment of experts and non-experts in the first semesters of the model curriculum, Inter-PTT appeared to be a worthwhile experiment. The implementation should help to clarify whether Inter-PTT is better suited to train the tutors more focussed and effectively, to familiarise them with their role as facilitators, and to increase their personal motivation for PBL as a form of teaching.

## 2. Project description

### 2.1. PTT concepts, trainers and participants of PTT

Trad-PTT and Inter-PTT were offered in 2012 and 2013 by a total of four experienced PTT trainers. All of them had the same teaching material (ppt presentations, handouts and 23 PBL cases – 3 non-medical, 20 medical). The evaluation of the PTT trainers of 2012 confirmed that all were equally competent, had PBL expertise, and were able to motivate the PTT participants.

Everyone, who was interested in PTT, was offered 4 to 6 appointments, however clinicians and non-clinicians were taught separately in Trad-PTT in 2012 (clinicians: 2 days; non-clinicians: 3 days). In 2013, there was only the two-day inter-PTT, which was interdisciplinary, i.e. at least one non-clinician was in each training group. The graduates of Trad-PTT (2012) were combined as Cohort 1, the ones from Inter-PTT (2013) as Cohort 2.

#### 2.2. Evaluation questionnaires

All PTT participants were asked to complete an evaluation questionnaire with three categories after the PTT. The Trad-PTT tutors received the evaluation questionnaire at the end of 2012, while the Inter-PTT tutors received them on the second day of training. The first section covered the tutors’ teaching experience and their motivation to participate, and the second section asked for the evaluation of the completed PTT. The third section addressed the PBL motivation of the tutors and the overall assessment of the PTT. For ratings, a 6-stage Likert scale was used (6=very good, 1=very bad).

#### 2.3. Interim evaluation and PTT revision

At the end of 2012, the questionnaires of 12 months Trad-PTT were evaluated to discuss didactic changes. The new concept (Inter-PTT) started in 2013 and also ran for 12 months.

#### 2.4. Follow-up survey

At the end of 2014, a follow-up survey of all tutors from Trad-PTT and Inter- PTT followed, i.e. 12- 36 months after they had completed their PTT. The first PBL experiences were recorded along with the subsequent assessment of whether the respective PTT had prepared well for PBL.

#### 2.5. PBL self-commitment

Since the evaluation in 2012 showed that only about 50% of the PTT tutors actually taught PBL afterwards, a self-commitment for all departments was introduced by the Vice Deanship of Studies and Education in 2012: For each PTT trainee, the department had to self-commit to teach at least two PBL groups in the following four semesters. The fulfilment of the commitment was assessed 12 months later by the vice deanship.

#### 2.6. Statistical evaluation

The statistical analysis was performed with SPSS (version 23). Categorical characteristics were presented as absolute and relative frequencies. According to the argumentation of Geoff Normann [16], the arithmetic mean values and the standard deviations were used despite their ordinal scaling for the descriptive representation of the Likert scale results. Confirmatory analyses for comparison of cohorts were performed using Mann-Whitney-U tests. P-values ≤0.05 (double-sided) were considered significant. No Bonferroni correction was performed.

## 3. Results

### 3.1. Characterization of the cohorts

235 tutors (cohort 1) completed Trad-PTT, 43% of which completed the evaluation questionnaire. 195 tutors (cohort 2) completed Inter-PTT, of whom 85.6% evaluated the training (see Table 3 (Tab. 3)). In comparing the professional background, 68.0% of cohort 1 came from medicine, including dental and veterinary medicine, 32.0% from natural sciences or humanities. 54.7% of the medical tutors came to from clinical disciplines, 45.3% from preclinical or research fields. The values were comparable in cohort 2. In cohort 1, 87.1% already had teaching experience (more than one answer possible); in cohort 2, 78.4% had teaching experience. Participation motivation was differentiated by participation upon own request, on the recommendation of colleagues or delegation by superiors (more than one answer possible). All those who had marked “delegation by superiors” were summarized as “involuntary”. In both cohorts, about half of the tutors involuntarily participated in PTT.

#### 3.2. Evaluation of the two PTT concepts

The comparison between Trad-PTT and Inter-PTT using the Likert scale is shown in Table 4 (Tab. 4). The following points turned out significantly better in the arithmetic mean for Inter-PTT compared to Trad-PTT: Understanding the PBL principle, the MSM structure and the tasks in PBL teaching. The increased training of PBL cases (medically and non-medically) in Inter-PTT was considered a significant improvement; The PTT trainers also received better evaluations. In terms of the interdisciplinary aspect, the assessment of team work and cooperative atmosphere improved. Also, the motivation to teach PBL was greater in cohort 2. The overall assessment of the training was significantly better for Inter-PTT than for Trad-PTT. All differences between Trad-PTT and Inter-PTT were highly significant (p<0.001).

In the relationship between teaching-experienced and inexperienced tutors, there were hardly any differences between the subgroups (88 experienced in cohort 1, 136 in cohort 2). The ratio of involuntary to the voluntary tutors was essentially constant (n=52 involuntary in cohort 1, n=106 in cohort 2).

#### 3.3. Results of follow-up surveys of both cohorts

The questionnaires were evaluated separately according to Trad-PTT and Inter-PTT (see Table 5 (Tab. 5)). The differences between both cohorts with regard to PBL motivation were low: All respondents were fine with this teaching format and liked to teach it. In the evaluation of the completed PTT, Inter-PTT performed significantly better in all categories: The graduates reported that they benefited from the PTT didactically and that the training prepared them well for the PBL lessons. On the question of being interdisciplinary (“PTT has facilitated contact with other subjects”), there were no differences between the PPT concepts.

#### 3.4. Results of self-commitment

The number of PTTs (see Table 6 (Tab. 6)) increased as a result of the introduction of the model curriculum. The introduction of self-commitment led to a reduction in PTT requirements, and still more than 100 new PBL tutors per semester were available. The number of PBL teaching departments remained constant with just under 60 of 114 departments, of which approximately half were clinical departments. The number of first-time PBL teachers (relative to all PBL tutors) has remained constant since the introduction of self-commitment. The request for self-commitment in 2013 showed that only individual departments did not comply with their obligations, because trained PBL tutors were no longer available due to expired contract, rotation or parental leave.

## 4. Discussion

In 2003, John Hattie’s meta-analysis shook the teaching world by pointing out that the personality of the lecturer has the greatest impact on the learning process [17]. In the medical field, clinical education conveys the doctor’s image optimally: The majority of students in the third year of study felt that observing doctors in their clinical activity was the most important role model for professionalism [18]. For PBL lessons, engaging tutors are needed who can handle feedback, are willing to question their approach, and thereby improve their teaching strategy [1]. Therefore, our goal was to teach PBL as a teaching method for future PBL tutors and to motivate them to promote the learning process in PBL as a facilitator, not as a frontal, teacher-centred process.

In the reform curriculum, recruitment of PBL-interested lecturers for the 27 PBL groups was achieved without any difficulties. PBL was preferably taught by clinicians, although some students missed the teaching of basic knowledge. In the model curriculum, PBL was supposed to take place for all students (maximum of 400 PBL groups per semester). Since many pre-clinical and clinical-theoretical internships were lost in favour of interdisciplinary internships, sufficient lecturers with interest in PBL were available. However, they were unsure whether they could competently mediate the clinical background of PBL cases.

Inter-PTT quickly confronted the tutors with clinical cases through the joint training of clinicians and non-clinicians. The clinicians were continually asked to explain terms and backgrounds and the interdisciplinary concept was quickly implemented by all participants. The high proportion of interactive formats increased the focus on the disciplinary boundaries. On the second day, each tutor had to present a clinical PBL case (steps 1-4) related to their own field (e.g. diabetes mellitus for biochemistry assistants). Non-clinicians, who mastered the basic knowledge of the case, were more focused on PBL steps and group dynamics. Clinical participants often fell into the role of the frontal teaching in cases of their own field of study, which was quickly noticed in external and self-observation and discussed in the feedback. This encouraged the discussion of possible strategies to implement the role of facilitator. The non-clinicians learned to incorporate theoretical knowledge with clinical relevance into the PBL case (e.g., lack of spatial vision in retinal detachment).

The interdisciplinary concept taught the tutors that in PBL background as an expert or non-expert is immaterial [19]. The term “expert” is discussed, because students initially perceive preclinical lecturers as experts [20]. In addition, students associated “expertise” with the idea that the PBL tutor possessed the science background of the case, not their social competence [21]. Many employees from clinical departments, which are exclusively active in research, are nevertheless evaluated by students as competent PBL tutors [22]. 

The PBL cases used on the second day were individually tailored to the tutors’ subject areas, so that they consider important aspects in the role of facilitator:

1. Incorporate all group members actively in the PBL process,
2. Relate the learning process to the PBL case,
3. Reveal the understanding of the students, and
4. Encourage students to evaluate information autonomously [23].

The tutors were repeatedly asked to try out different learning steps as a facilitator in the PTT (for example, to slow down dominant participants in order to encourage restrained participants, to support unusual visualization techniques, and to develop value-neutral formulations).

Most strategies to mediate PBL successfully focus on group dynamics and promote critical thinking [12]. Since the process of learning support depends on the tutor’s approach, he should be critically recapitulated on a regular basis [24], since teacher training is an important prerequisite for good teaching. The share of PTT graduates with no teacher experience rose from 12.9% (cohort 1) to 21.6% (cohort 2) signalling that more young employees were sent to PTT. Self-commitment could also have forced departments to invest more in the long term. The PBL tutors knew that they would be used for PBL in the successive semester, which might have increased the concentration during training.

In Cohort 2 the proportion of involuntary participants increased slightly. These tutors found the training an annoying commitment; nevertheless, Inter-PTT succeeded in capturing these openly involuntary participants. The difference in the scores between voluntary and involuntary tutors was low, as Kuhnigk also noticed during the didactic training [25]. Both groups rated Trad-PTT as good, but Inter-PTT significantly better. 

Assessment of the evaluation questionnaires showed that Inter-PTT significantly improved the needs of the tutors. Five articles on the theory of group dynamics and the pedagogical basis were emailed to the tutors two weeks before the PTT. Although only a minority read them before the training, the processing of two PBL cases on the first day provided questions on the theoretical background and the role of the PBL tutor. Thus, the self-study period was actually used for the processing of the learning goal and the literature study. 

On the first day, the introduction to the structure of the model curriculum took place because the tutors should understand the importance of PBL in a reform curriculum [15]. The teaching of the basic idea of this curriculum also supported the interdisciplinary learning process at PTT. Collaboration in the PTT group was difficult on the first day because most of the tutors initially concentrated on presenting their (medical) competence. It was only after interactive case training that the group began to develop teaching strategies together.

The cohort 2 participants confronted the processing of a clinical and non-clinical PBL case with unusual problems, which activated prior knowledge and motivated them to develop strategies for long-term retention [26]. The tutors quickly realized that they assumed the student perspective, and a “we” feeling emerged beyond the boundaries of the respective field. The learning goal formulation encouraged a critical discussion of operator verbs that forced the definition of the intensity of operators.

In Inter-PTT, an open group discussion replaced the theoretical input in difficult situations: Each tutor received a typical situation (for example, a student is talking continuously) and was supposed to reflect on it:

1. Have I already experienced this situation?,
2. How did I react?,
3. What would have caused a different reaction?

Initially, the coaches reported their own sub-optimal situation, then the tutors exchanged their experiences. Almost all of them remembered teachers from their school or university, who impressed them with how they handled critical situations. Other responses were discussed and the tutors realized that everyone needs to develop their personal solution strategy. Although many instructors had years of teaching experience, they were confronted with situations in PTT role playing where their usual action would have interrupted the PBL process. This led to the exchange and testing of other reaction possibilities. In presenting medical PBL cases, the group’s intensive feedback helped each tutor develop his own toolbox of facilitation strategies. 

The Inter-PTT concept with less theory and more interactive teaching phases brought the participants closer together. They had less inhibitions to ask for help from experts, which initiated a collaborative learning atmosphere [13]. The interdisciplinary idea was supposed to be conveyed to the students later as a positive role model in order to promote self-organized learning. However, the interdisciplinary exchange was lost in PBL practice, as the follow-up survey showed. 

One limitation lies in the evaluation participation: The regression increased sharply from Trad-PTT to Inter-PTT (43% versus 85.6%). This could be due to the fact that cohort 1 was questioned at the end of the year, whereas cohort 2 was evaluated directly after the PTT. In the follow-up survey, the regression of both cohorts was similar, although cohort 2 returned the questionnaire more frequently. The follow-up survey also made it clear that the basic setting levelled off compared to PBL but does not change significantly.

## 5. Conclusion

Overall, Inter-PTT was able to successfully communicate the PBL process to clinical and non-clinical participants. The theoretical input was reduced, which shifted the focus on interactive learning and case training. The critical discussion of PBL cases gave the tutors an in-depth look at their role as facilitator and provided room for the development of their own teaching strategies for PBL. The self-commitment led to the continuous recruitment of PBL tutors, even if they came to the training involuntarily. According to Hattie’s observation, Inter-PTT was highly effective in exciting the tutors about the PBL concept and preparing for PBL. This format will form the basis for further development of training concepts for PBL tutors.

## Acknowledgements

Thanks to Jörg Pelz, who has done a lot for PBL; he passed away in 2014.

## Competing interests

The authors declare that they have no competing interests. 

## Figures and Tables

**Table 1:**
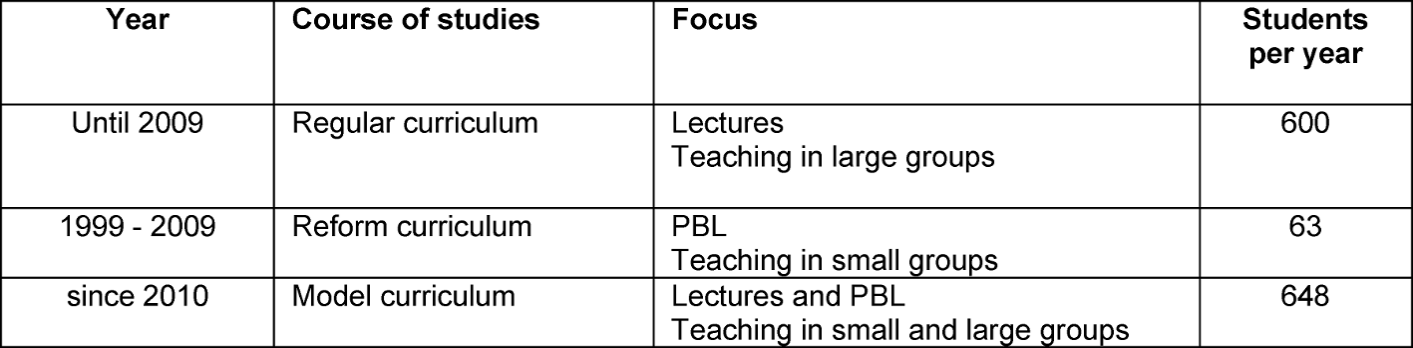
Characterization of human medical studies at the Charité University Hospital in Berlin

**Table 2:**
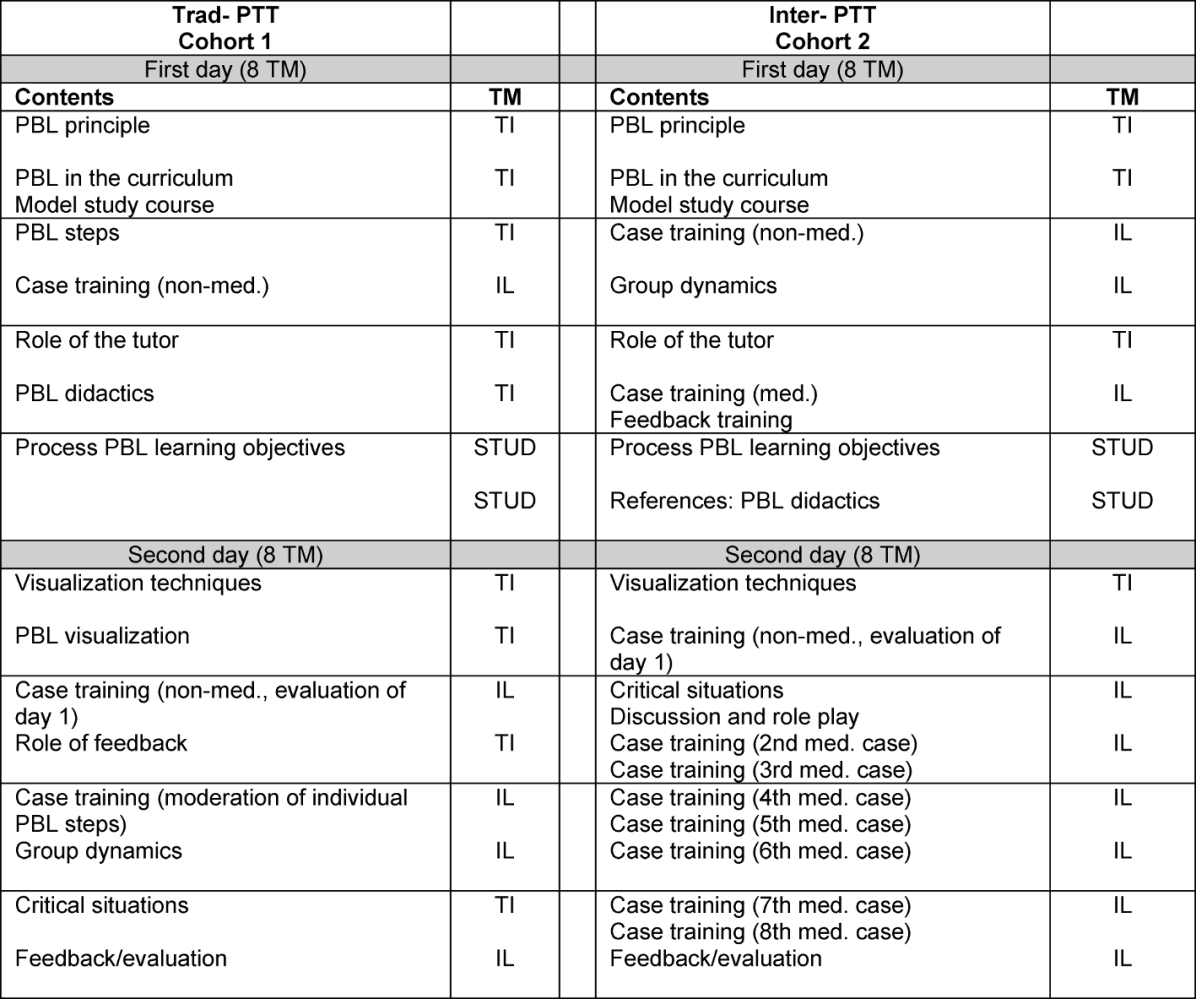
Conceptual comparison between traditional PTT (Trad-PTT) and interactive PTT (Inter-PTT). The training modules (TM) occur as theoretical input (TI), interactive lessons (IL) and self-study (STUD). For PBL case training, medical (med.) and non-medical (non-med.) PBL cases were used.

**Table 3:**
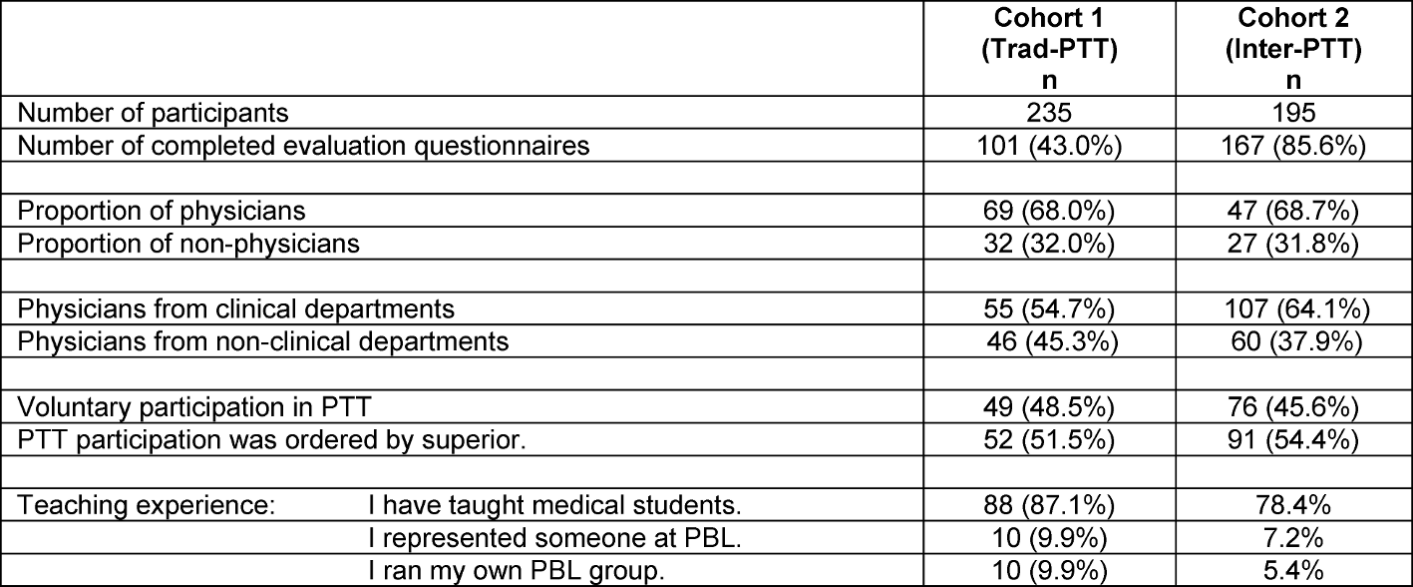
Characterization of both PTT cohorts. Cohort 1 completed the traditional PTT, cohort 2 the interactive PTT (Inter-PTT).

**Table 4:**
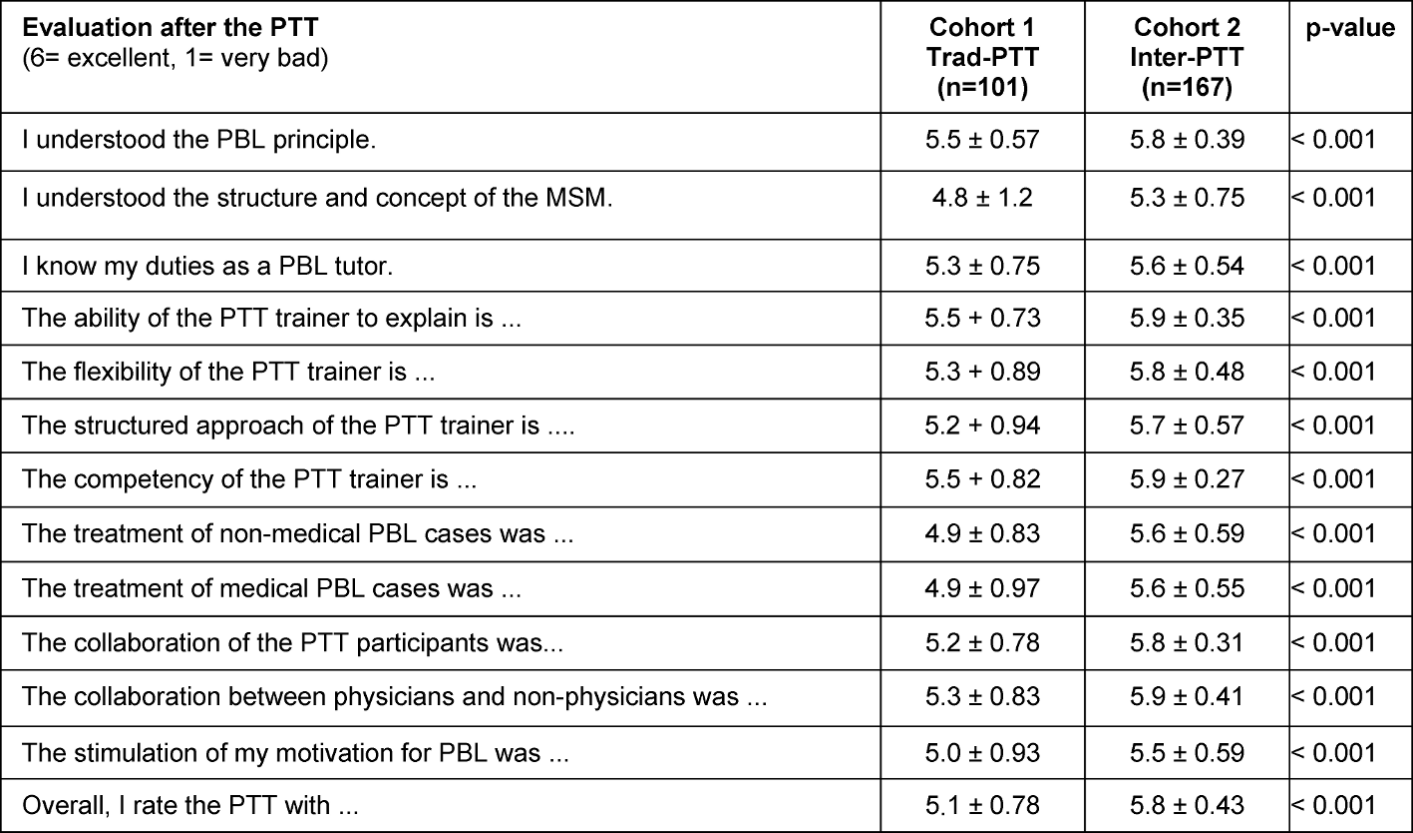
Evaluation comparison between traditional PTT (Trad- PTT) and interactive PTT (Inter-PTT) after training. The mean values are based on a 6-stage Likert scale.

**Table 5:**
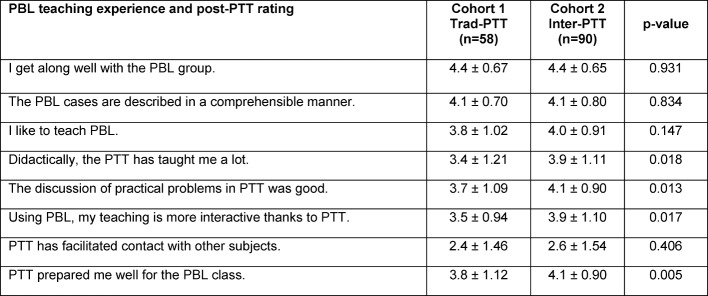
Results of follow-up surveys of both cohorts. Respondents completed either the traditional PTT (Trad-PTT) or the interactive PTT (Inter-PTT).

**Table 6:**
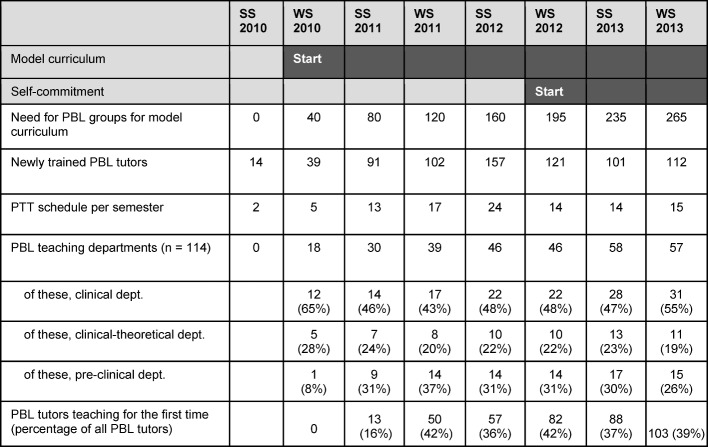
Results of PBL self-commitment. The introduction of self-commitment took place in the winter semester 2012 (WS = winter semester, SS = summer semester, dept. = department).
